# Development and Implementation of the Portable Operating Room Tracker App With Vital Signs Streaming Infrastructure: Operational Feasibility Study

**DOI:** 10.2196/13559

**Published:** 2019-08-05

**Authors:** Matthias Görges, Nicholas C West, Christian L Petersen, J Mark Ansermino

**Affiliations:** 1 Department of Anesthesiology, Pharmacology & Therapeutics The University of British Columbia Vancouver, BC Canada; 2 Research Institute BC Children’s Hospital Vancouver, BC Canada; 3 ESS Technology Inc Kelowna, BC Canada

**Keywords:** communication systems, patient monitoring, user-centered design, human factors, anesthesia

## Abstract

**Background:**

In the perioperative environment, a multidisciplinary clinical team continually observes and evaluates patient information. However, data availability may be restricted to certain locations, cognitive workload may be high, and team communication may be constrained by availability and priorities. We developed the remote Portable Operating Room Tracker app (the *telePORT* app) to improve information exchange and communication between anesthesia team members. The *telePORT* app combines a real-time feed of waveforms and vital signs from the operating rooms with messaging, help request, and reminder features.

**Objective:**

The aim of this paper is to describe the development of the app and the back-end infrastructure required to extract monitoring data, facilitate data exchange and ensure privacy and safety, which includes results from clinical feasibility testing.

**Methods:**

*telePORT*’s client user interface was developed using user-centered design principles and workflow observations. The server architecture involves network-based data extraction and data processing. Baseline user workload was assessed using step counters and communication logs. Clinical feasibility testing analyzed device usage over 11 months.

**Results:**

*telePORT* was more commonly used for help requests (approximately 4.5/day) than messaging between team members (approximately 1/day). Passive operating room monitoring was frequently utilized (34% of screen visits). Intermittent loss of wireless connectivity was a major barrier to adoption (decline of 0.3%/day).

**Conclusions:**

The underlying server infrastructure was repurposed for real-time streaming of vital signs and their collection for research and quality improvement. Day-to-day activities of the anesthesia team can be supported by a mobile app that integrates real-time data from all operating rooms.

## Introduction

### Background

Pediatric perioperative care can be a hectic and stressful work setting, in which a multidisciplinary team of clinicians (anesthesiologists, surgeons, nurses, and other health professionals) continually observes and evaluates patient information [1-3]. Efficiency and patient safety in the procedural suites depend on a well-functioning team [1,2]; however, data are often limited to certain locations, cognitive workload can be high, and team communication constrained by availability and differing priorities [4,5]. Even experienced teams face challenges of physical separation and the need to locate each other by pager and phone [6].

To address this problem, our team of clinicians, engineers, and computer scientists developed a remote Portable Operating Room Tracker app (the *telePORT* app) to improve information exchange and communication between anesthesia team members. It aims to combine a real-time feed of physiological waveforms and vital signs from the operating rooms (ORs) with messaging, paging and reminder functions.

### Clinical Setting

BC Children’s Hospital (BCCH) is a tertiary pediatric medical center in which approximately 11,500 children per year undergo general anesthesia for surgery, dental procedures, endoscopic investigations, medical imaging, and other interventions. On any weekday, there are approximately 12-15 anesthesiologists covering a variety of locations, including the core 8 ORs, the oncology suite, X-ray, computed tomography (CT) scan, magnetic resonance imaging (MRI) and ultrasound rooms, and a burn treatment room.

Their work is supported by a small team of anesthesia assistants (AAs). These are allied health professionals, with backgrounds in respiratory therapy, who work with the attending anesthesiologists to optimize the safety of the anesthetized children by ensuring that equipment is maintained and readily available, and by providing extra assistance when needed. They are a limited resource, as only 3-4 AAs are available at any time and they are often tied up helping start complex procedures, such as cardiac, neuro-, or spine surgery. Each AA typically supports more than one anesthetic location and ideally needs to be able to monitor the status of multiple patients. They must be responsive to requests for assistance and, if possible, maintain ready awareness of the situation that precipitated the call for help.

To request an AA, anesthesiologists relied on a numeric, one-way, phone-based paging system, which could be cumbersome to use from the anesthesia workstation, provided no feedback about AA availability or the urgency of the message, did not allow two-way communication, and did not allow delegation of tasks if the AA was unavailable. Nonetheless, the paging approach was well established, as it was secure and reliable and was still in use at BCCH when we began the *telePORT* project. It gave us the opportunity to improve teamwork through two-way, secure communication and information to improve the situational awareness of the AAs.

### Technical Setting

Physiological multiparameter patient monitors in ORs and intensive care units (ICUs) gather data from multiple sensors and diverse formats. These include, for example, physiological waveforms like the electrocardiogram (ECG), from which numeric data such as heart rate and alarms (eg, bradycardia [low heart rate]) are derived. Most ORs and ICUs are multibed environments, in which individual patient monitors are connected to central monitoring stations that support event recording, centralized printing and remote alarm monitoring. These central stations are immobile and have limited proprietary interfaces, which impose a barrier to secondary use of their data. Yet significant opportunities exist if these data can be made available on a secure mobile platform, ideally augmented with decision support systems.

These data gathered from vital signs, covering a wide range of settings and procedures, are valuable for research, quality improvement (QI), and review of undesired clinical events or outcomes that occur during normal clinical care.

At BCCH, multiple vital sign parameters are monitored in the OR (including the postanesthetic care unit) and pediatric ICU, and they are captured automatically and stored in a central station server. Yet, this mass of data is routinely discarded after 2-3 days, with only small samples manually or automatically transcribed in infrequent intervals (typically at 5 min, 15 min, 1 hr, or 4 hrs) into the patient's medical record. To overcome this limitation, we obtained ethical approval to collect and store all vital signs data from children in the OR in 2009 and in the ICU in 2011 for research and QI purposes.

*LambdaNative* is an open source software framework, designed for developing medical apps, that promotes deterministic, robust and correct code [7]. It is based on the portable Gambit Scheme programming language [8] and provides a flexible cross-platform environment for developing graphical apps on mobile devices as well as medical instrumentation interfaces on embedded platforms [7]. It has been used to create a diverse range of applications, from a mobile data collection tool for a multicenter preeclampsia trial in low resource settings [9], to mission critical, embedded drug delivery as part of a closed-loop anesthesia system [10]. Importantly, we identified strategies to improve medical device data exchange using a data broker concept [11].

### Aim of Study

In this article, we describe the development of the *telePORT* mobile app using user-centered design. We also outline the back-end infrastructure (VitalNode) required to extract monitoring data, facilitate data exchange, and ensure privacy and safety. This section includes some technical details that may be helpful to some readers (in particular those who do not have an Anesthesia Information Management System).

## Methods

### The Main App Features

**Figure 1:**
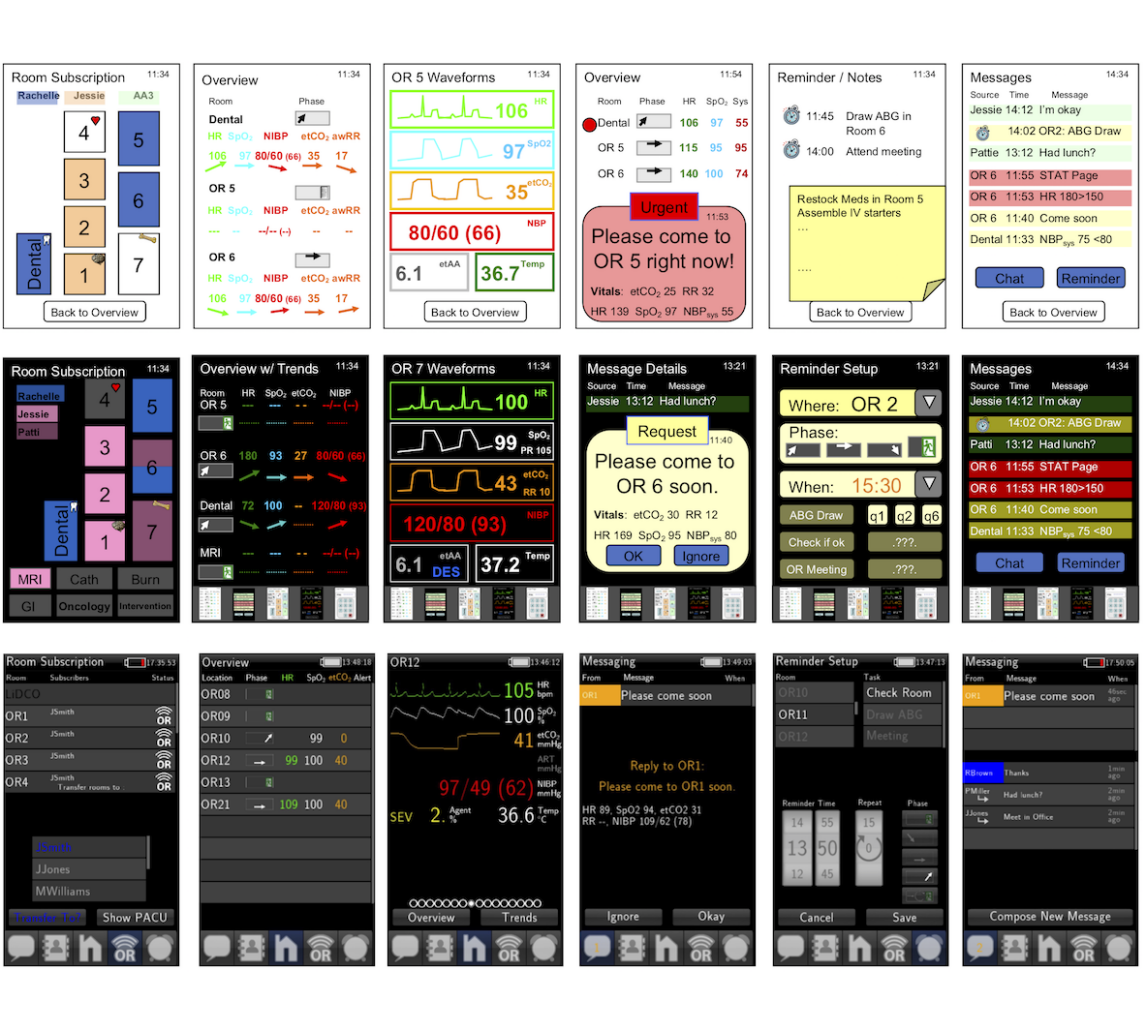
Progression of the *telePORT* app from initial concepts (top row), to intermediate prototype re-designed after user feedback (middle row), to final app (bottom row). The app features shown are: room subscription screen (first), overview screen (second), waveform/numeric detail screen (third), help request screen (fourth), reminder setup (fifth), and messaging overview screen (sixth).

The purpose of *telePORT* is to improve information exchange and simplify communication between anesthesia team members. We focused the design on five key features, all of which could be reached at any point using a menu navigation bar at the bottom of the screen (Figure 1).

#### Overview Screen

This screen provides basic information about the patient monitoring locations to which the user is subscribed. The information displayed includes an anesthetic phase indicator and three vital signs: heart rate (HR), oxygen saturation (SpO_2_), and end-tidal carbon dioxide concentration (etCO_2_). A messaging icon displays the number of unread notifications for each location (Figure 1). Secondary screens show either waveforms and additional numeric values, in a design similar to the patient monitor layout, or a 30-minute trend screen, which can be obtained by selecting a location (Figure 1). The monitoring screen allows swiping to switch quickly between screens and features a full screen mode which can be reached by changing the orientation of the device to landscape. This was added late in the development cycle as a frequently requested feature.

#### Messaging Screen

This screen provides a combination of person-to-person chat and a system to receive requests for help through a button pressed on the patient monitor in an OR. Both types of message are located in two separate areas of the screen with automatically generated messages (requests for help from the OR and notifications from reminders) in the top panel and sent messages in the bottom panel (Figure 1).

Requests for help from the OR are initiated by the anesthesiologist pressing the physical Snapshot button on the patient monitor, with two presses in a 4 second window initiating an urgent (STAT) call. These requests prompt the display of a popup window. If selected from the popup or system messaging list, further information is displayed including the origin location, message urgency (“Please come soon” or “STAT Request”), a selection of vital signs including HR, SpO_2_, etCO_2_, respiratory rate (RR), and blood pressure (NIBP). The app allows a response of Okay or Ignore (Figure 1). If the user presses Ignore or fails to confirm the message within 5 min, the alarm is escalated to all other users logged into the system. Urgent (STAT) messages are sent to all users, regardless of the locations to which they are subscribed. Providing vital signs with the request allows the user receiving the message to obtain some information about the situation before arrival, in an effort to raise situational awareness [1,12,13].

For the person-to-person messaging system, we support quick texts, with 10 options including messages such as “Yes,” “No,” “Can you help?,” “Meet in office,” and so on, as well as a regular two-person chat system with an on-screen keyboard. The quick text list was developed by asking anesthesia assistants for the most commonly used texts rather than through something like a self-learning approach. A special user (ALL) allows messages to be sent to all users, but responses will only reach the sender as group chat was not implemented.

#### Phonebook Screen

This screen lists frequently used phone extensions, such as each OR, the biomedical engineering department and pager numbers, and can be edited within the app. We envisaged adding a Voice over internet protocol (VoIP) feature to this screen but it was never completely implemented.

#### Location Subscription Screen

This screen shows which locations a user is subscribed to and also the other team members requesting information from that location. The user can subscribe to, or unsubscribe from, each of the monitoring locations and subscriptions can be delegated to other users (the recipient must accept the transfer). The initial design called for a visual map, with icons for cardiac, dental, neurosurgical, orthopedic and other ORs, to allow users to select locations based on their physical proximity. It was later modified into a simple list as it is faster to use, and as assignments are more frequently based on the case complexity and not physical proximity.

#### Reminder Screen

The fifth screen allows the user to set up reminders based on time or anesthetic phase (Figure 1). For time-based reminders, the user selects time (using scrolling wheels for hours, and minutes in 5 min increments), reminder frequency (none, 15, 30, 60, 90, 120, and 300 min), and tasks (“Check Room,” “Draw ABG,” and “Meeting”). For reminders based on anesthetic phase, only the location, task, and anesthetic phase symbol are selected. A special empty room cycling mode allows repeated reminders whenever the room phase changed to empty until the end of the day. It was intended for use in high turnover lists, such as endoscopies, where restocking is performed frequently throughout the day. Repeated reminders were a requested feature to support the arterial blood gas (ABG) workflow, in which the anesthesiologist periodically (eg, every 2 hrs) draws a blood sample, which the anesthesia assistant then takes to the point-of-care analysis device for processing before returning with the results to the OR.

### Development of the VitalNode Server Component

The multiparameter patient monitors transmit real-time data to a central station via a dedicated ethernet network. To overcome limited access to the proprietary central station interface, we intercepted raw ethernet data packages directly.

**Figure 2:**
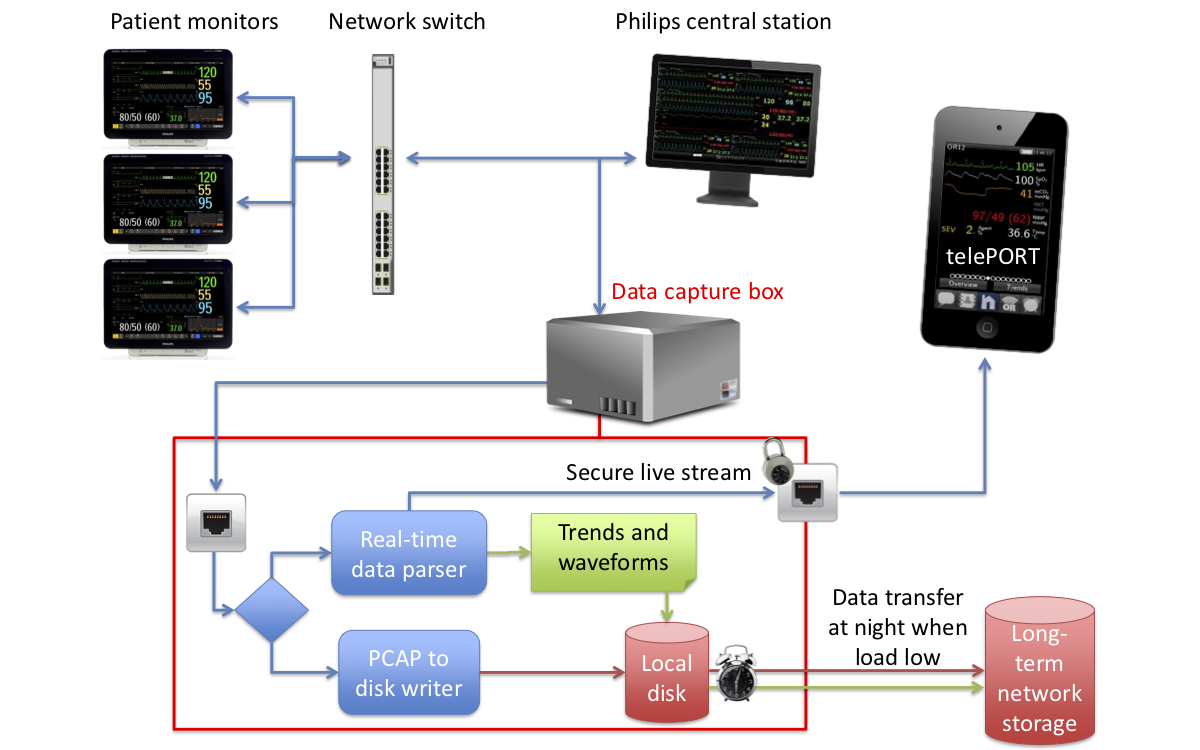
Overview of the data collection system. Data from the patient monitors is captured by the VitalNode box before it flows through the switch to the central station. This data is parsed, encrypted, and made available to the *telePORT* app for real-time use, and also stored in both parsed (trends and waveform CSV files) and raw formats. PCAP: packet capture.

We initially used a passive network tap on the transmitting wires of the ethernet cable connecting the main OR and ICU switch aggregating data from the monitor to the central station. These were connected to a network port which was set to monitoring (promiscuous) mode to collect the received network traffic. In a later iteration we bridged two network ports, as this solution was easier to implement on the updated 100-megabit full duplex network which automatically assigned receiving or transmitting wire pairs. The device doing this work was called ‘VitalNode.’ A hybrid approach was taken for the data, with the raw data stored but also parsed in real-time for processing (Figure 2).

### Real-Time Data Parsing

Real-time parsing of the network traffic involved decoding the data frames of the Philips and GE or Datex monitor communication protocols. The Datex protocol was published accurately in the programming guide, whereas the Philips protocol had undocumented header data structures that were decoded using empirical analysis. For performance reasons we chose to employ a kernel level filter, restricting parsing only to packages of types we knew the parser could analyze and which contained data useful for our purposes.

The VitalNode app performed several tasks, including: 1) extracting key physiologic trend data from all available vital signs (eg, SpO_2_, HR, temperature) at 10 sec (Datex monitors) or 1 sec time resolution (Philips monitors); 2) parsing alarm data; 3) parsing relevant physiological waveform data (eg, ECG, plethysmograph) at resolutions between 25 and 500 Hertz depending on the vendor and variable; and 4) extracting demographic information (rarely entered).

These data were written to disk in comma-separated values (CSV) format for later use, separating the 5 or 10 second interval trend files of all potential variables that could be captured. Then, waveform files were written if a sensor was sending valid data, to simplify data extraction and processing. Data was kept in memory for near real-time data access in streaming applications. VitalNode also stored the messages exchanged in the *telePORT* app, both by provider (to allow person-to-person communication), and by monitor location (to store system generated messages that were available to all subscribers of a location).

The app aggregated 30 mins of trend data (in 10 sec resolution), for ease of display. It also applied some simple data processing, including an anesthesia phase indicator. This used a simple 4-state machine, based on the presence or absence of valid values from three commonly used sensors (SpO_2_, HR, and etCO_2_, using 30 sec averages). This allowed us to determine if the case had started (induction of anesthesia, one sensor present), was in the middle of the case (maintenance of anesthesia, all three sensors present), was nearing the end of the case (emergence from anesthesia, one sensor off), or if the room was empty (no sensors reporting valid data). To avoid problems during cardiopulmonary bypass, zero values for etCO_2_ were considered valid while empty or error states were deemed invalid.

### Raw Data Storage

We used raw network package capture (PCAP) files in 10-min chunks, compressed using bzip2, for two reasons: 1) these files use disk space efficiently; and 2) this approach allowed us to capture data elements not implemented in the data parser and to recover from potential errors in the parser code.

### Development of the telePORT Client Component

A user-centered design process was used [14]. Initially, three AAs were shadowed during two of their day shifts to identify their information needs, communication and reminder strategies, and task planning approaches. Combined with semi-structured interviews, these data were used to create a modified work-domain analysis [15,16], which established a hierarchical model of the domain and allowed specification of the app requirements. Next, we conducted a participatory design of the mobile app [17-19], in which we sought frequent feedback on design features, initially using mockups developed in PowerPoint (Microsoft, Seattle, WA), and later in partially working prototypes presented on an iPod touch (Apple, Cupertino, CA; Figure 1).

We used *LambdaNative* [7] to rapidly develop a mobile app targeted to run on a small mobile tablet or smart phone, such as a 4th generation iPod touch, with an effective screen resolution of 480x320 pixels. Initial development was performed on a Linux machine with only the iOS building process performed on Mac, thus highlighting one advantage of the cross-platform capabilities of the *LambdaNative* environment. The prototype components were implemented, underwent quick periods of usability testing by the AAs as well as research team members, and then were modified according to the feedback obtained. The app was deployed on iPod Touches using an Apple developer provisioning profile. A modified version (without continuous background processing and connectivity) is available on the Apple App store [20].

### Evaluation Approach

With approval by the University of British Columbia Children’s & Women’s Health Centre of British Columbia Research Ethics Board (H11-01785) and written informed consent, we established the baseline workload of the AAs using a hybrid approach: we used a step counter to obtain a surrogate for distance travelled, as well as daily logs of the number of pages received and the urgency of those requests. We originally planned to repeat these after the *telePORT* app was implemented, but the data was highly variable and data collection was not always comprehensive as users considered it too onerous, so we decided not to.

To evaluate the use of the *telePORT* app, we used both formal and informal feedback from AAs by email or verbal messages and quantified usage patterns using *VitalNode*-based data logging. Time stamped data of user logins, logouts, disconnects (loss of client connections exceeding 60 secs), navigated screens, message sender and recipients, and system-generated pages were recorded in CSV format on the *VitalNode* server. Data were parsed using R (R Foundation for Statistical Computing, Vienna, Austria), and plotted for analysis. We used battery usage as a surrogate for device usage (it being removed from the charging station for use) and explored message frequency and number of pages quantitatively. Cytoscape (Institute of Systems Biology, Seattle, WA) was used in an attempt to map navigational flow by creating bidirectional graphs of app screen log sequences.

## Results

### Baseline Step and Pager Tracking

In total, 27 data sheets were completed from October to December 2011 (an estimated response of 10-15% of shifts worked). The median (interquartile range [IQR]) number of steps was 6426 (IQR 5163-8910) per shift, or 627 (IQR 451-784) per hour. During this time the AAs reported 34 pages, which is a median of 0 (IQR 0-2.5) per shift, with the reasons for each including equipment in 12/34 (36%) cases, clinical in 14/34 (41%) cases, and administrative in 8/34 (24%) cases.

### Battery and System Usage

An early version of *telePORT* drained the iPod touch (4th generation, iOS 5.1, screen switched off) in 6-7 hours, which was insufficient for clinical use. Code was optimized to lower central processing unit (CPU) usage, achieving average battery drainage of 9.04% per hour, which allowed for 11 hours of battery life between charges.

**Figure 3:**
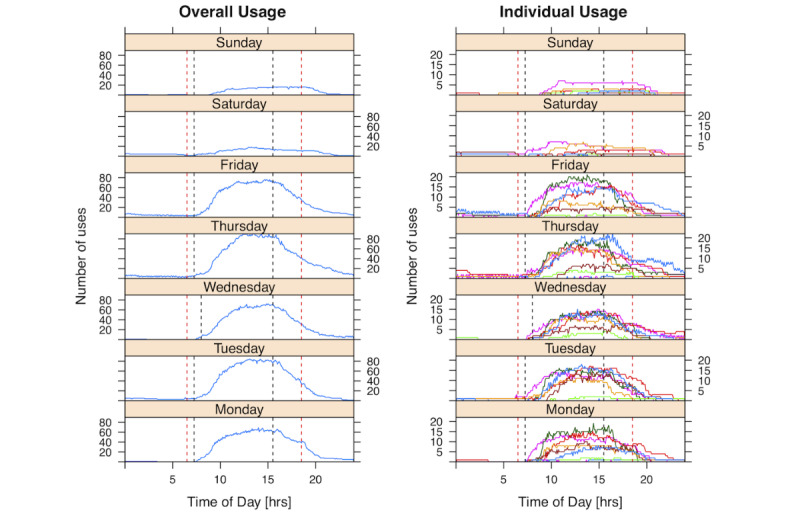
Usage of the *telePORT* app, split by weekday, over an 11-month period (48 weeks). Left panel shows overall usage; right panel has data split by user. Times are grouped in 5-minute increments. Overlaid are operating room core times using black dashed lines and typical user shift hours using red dashed lines.

During the evaluation, *telePORT* was used exclusively by the AAs (not by the anesthesiologists or Anesthesiologist in Charge). Data recorded on the 337 days between January 7, 2013 and December 9, 2013 were used to analyze usage, as this period coincided with peak usage and was available continuously. Battery drainage (as a surrogate of app use) showed usage patterns that overlapped with expected use, primarily during weekday OR hours, starting at 7h, with peak usage between 9h and 17h, and continuing until about 19h (Figure 3).

While usage varied widely during the evaluation period, with a median (IQR [range]) of 1.45 (1.0-2.0 [0.01-3.7]) devices in use each day, there was some continual decline in usage (–0.3% of usage per day) towards the end of the observation period. This decline was partly attributed to network communication issues reported by users but might also indicate workflow integration issues.

### Communication Flow and System Navigation

**Figure 4:**
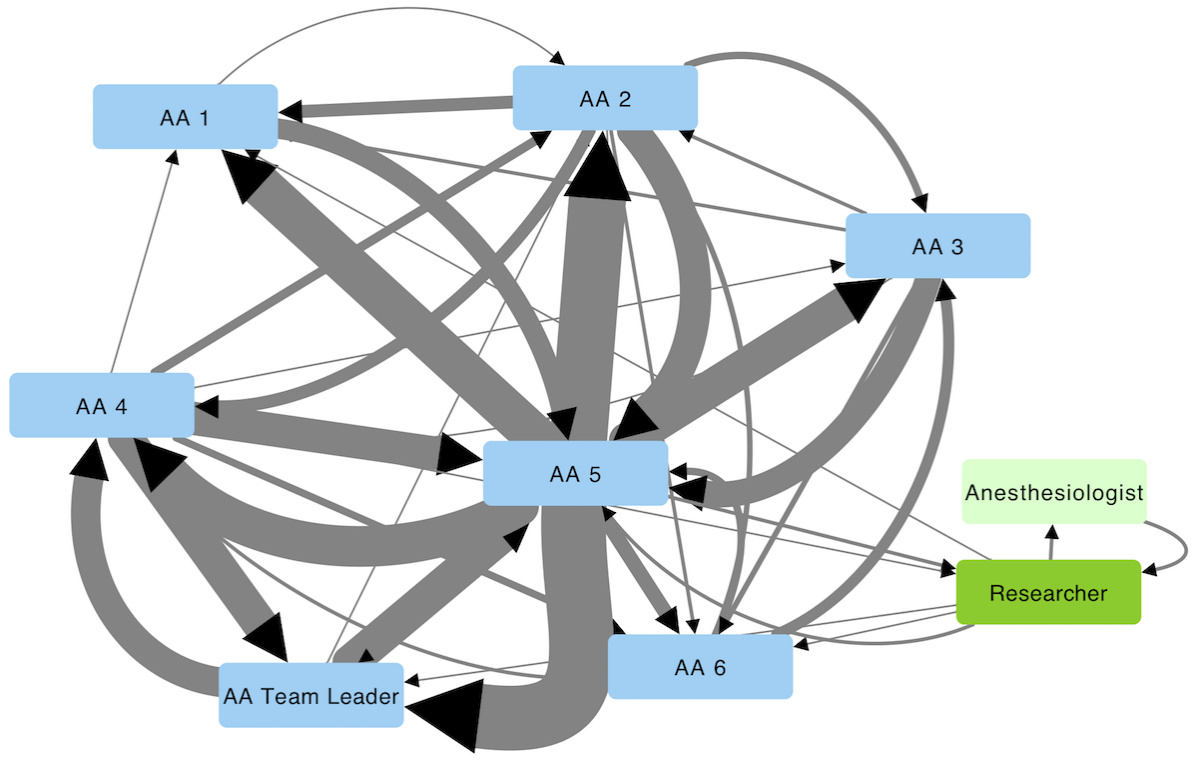
User communication pattern within the *telePORT* app. Blue nodes represent the anesthesia assistants and green nodes are other roles. The width of the connections is proportional to the number of messages sent. Black arrows indicate the receiver of the message. AA: anesthesia assistant.

**Figure 5:**
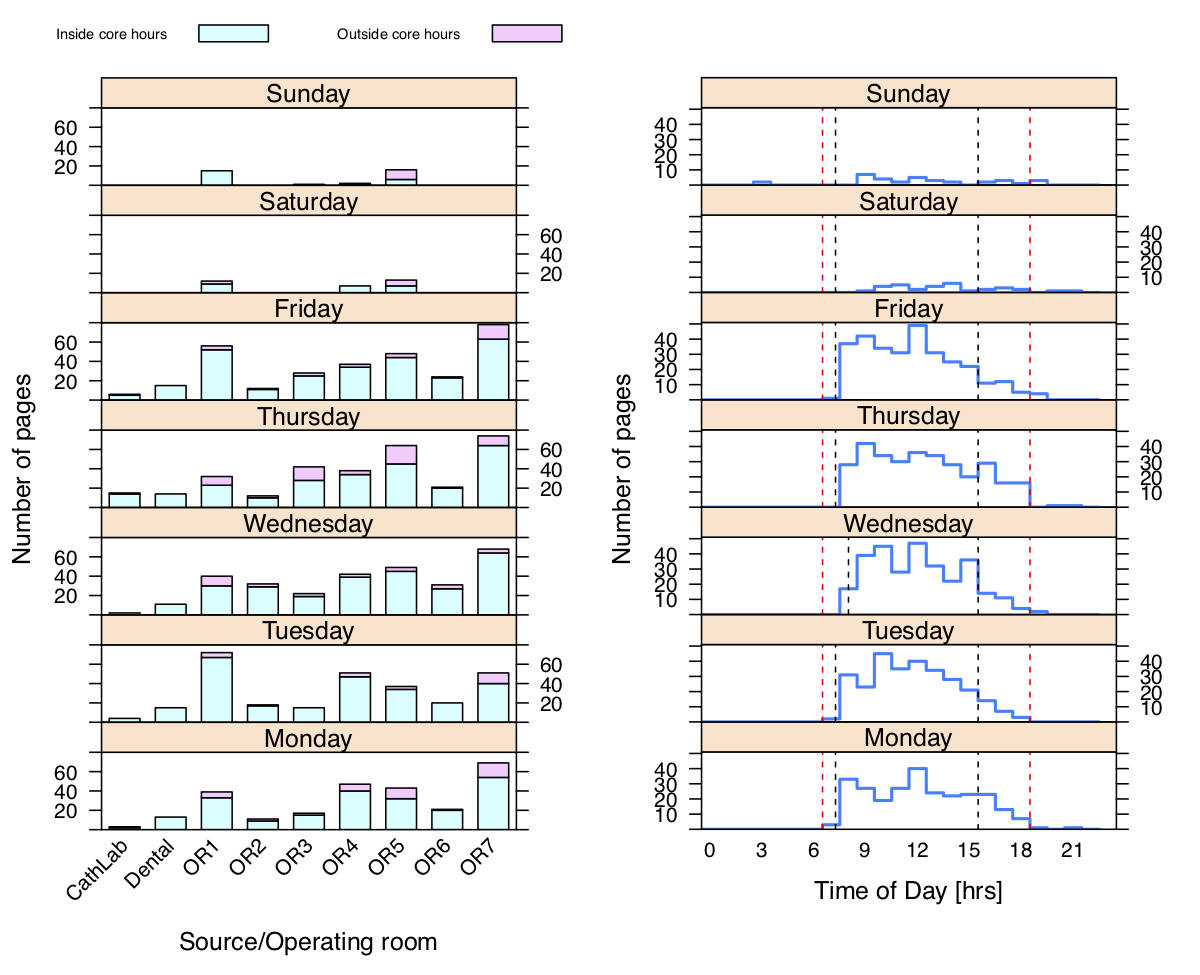
Paging feature utilization, split by weekday. Left panel shows paging usage by location, with those occurring inside core operating room hours indicated in mint and those outside these hours in light purple; right panel has data by time of day. Overlaid are operating room core hours using black dashed lines and typical user shift hours using red dashed lines.

A total of 327 messages (approximately 1/day) and 1528 requests for help (approximately 4.5/day) from an OR were recorded. Messaging pattern analysis found communication flow centered on one AA (AA5), who was not the team leader (Figure 4). The distribution of requests for help showed that OR7 (spine surgery) outnumbered other locations, but there was also a discernible pattern in OR1 which peaked on Tuesdays and Fridays (typically neurosurgery). Timing distributions did not show any surprising patterns, other than a peak number of messages in the middle of the day when new cases started (Figure 5).

The most commonly used features were Messaging (30%), Overview (20%), Pages or Alerts (19%), and Waveforms (14%). The integrated Chat screen was rarely used (2%), indicating a preference for using the 10 quick texts over free text communication. As the navigation bar allowed instant navigation between the five main features, only subscreen navigation shows signs of directed navigational patterns (eg, going from Reminder setup to Reminders).

## Discussion

### Main Findings

We developed the *telePORT* app to improve information exchange and communication between anesthesia team members, including messaging, help request (paging), and reminder features. In addition, *telePORT* provided a real-time feed of waveforms and vital signs from the ORs. Following development of the app’s user interface with user-centered design principles, *telePORT* was used by AAs at BCCH over 11 months to support their normal day-to-day activities. There were more help requests from anesthesiologists in the ORs (approximately 4.5/day) than messages between team members (approximately 1/day), but passive OR monitoring was also used frequently (34% of screen visits). Intermittent loss of wireless connectivity led to a steady decline in usage (–0.3%/day) and the project was eventually halted. The underlying server infrastructure has proved extremely valuable and continues to automate the collection of OR and ICU data for research and QI purposes.

### Team Communication and Situational Awareness

Utilization of *telePORT* was higher on the paging (request for help) functionality than when being used to exchange information between AAs, suggesting that anesthesiologists appreciated the simple way to request an extra pair of hands. Data also indicated that passive OR monitoring was common. This likely increased the AAs’ situational awareness, which may have preempted additional use of the paging function.

There are concerns about the use of smartphones in the OR, primarily around the potential for spreading infections, distraction for anesthesia team members, and interference with medical equipment [21]; however, they can improve team communication and provide an important learning tool [21]. Apps in the critical care domain are emerging [22], including tools like the Nurse Watch App which provides real-time vital sign monitoring, alarm notifications, and reminders [23], as well as a microblog messaging platform that synchronizes with a patient’s electronic health record and provides a forum for developing and sharing care plans [24].

In the perioperative setting, the VigiVU app (Vanderbilt University) [25] provides anesthesia care providers with notifications of vital sign deviations, changes in patient location, facilitates team communication and provides high quality video views into each OR. This system was developed and implemented by a much larger team and is more sophisticated than *telePORT*. To allow other researchers to learn from our lessons and continue development of both *telePORT* and VitalNode for use in other institutions, their source code has been made freely available (under a Berkeley Software Distribution license) in the LNhealth Github repository [26]. Perhaps the biggest accomplishment of the *telePORT* development process was the facility to live-stream vital signs to other devices for data collection in clinical research and QI projects. VitalNode, similar to the system in use at the University of Michigan [27], has facilitated use of vital signs data in a range of research and quality improvement studies at our institution [28-31].

### Implementation Issues: Connectivity and Battery Life

The main feasibility challenge was repeated problems with the hospital’s wireless area network. This led to a decline in use and eventual discontinuation of the project. The signal strength was deemed sufficient when tested, even in the presence of large concrete walls, but the problem with loss of connectivity (or, specifically, the failure to reobtain an internet protocol [IP] address from the dynamic host configuration protocol [DCHP] server) could not be overcome with the hospital network. Our ability to debug the problem was limited and we were unable to ensure that network features that facilitated handover (such as Institute of Electrical and Electronics Engineers 802.11k and 802.11r) were enabled on all access points. It may also have been a limitation of the mobile device we used (the iPod Touch). Had we used a cellular network–enabled device with a data plan we might have used Apple Push Notifications, which has been shown to be a particularly reliable mode of message exchange, even better than regular paging networks [32].

A similar limitation was noted by the developers of VigiVU, who required continued use of traditional pagers as connectivity suffered briefly when switching between certain wireless access points. While seamless handover had improved, it still required an institutional commitment to upgrade and maintain its wireless network infrastructure [27].

During the design stage, there was concern that the iPod Touch battery would not hold sufficient charge to support use during an entire shift. This proved to be a smaller problem than we feared, with batteries draining approximately 9% per hour, enough to last a typical shift. A similar finding was observed with the VigiVU app, in which the battery life left at the end of an 11-hour work day (from full charge) was around 25% [25].

### Mobile Apps and Decision Support Systems

The *telePORT* app extracts data directly from the OR patient monitoring system, but in some institutions, it is possible to use data extracted from an anesthesia information management system (AIMS), which provides for near real-time clinical decision support (CDS). For example, the use of the Smart Anesthesia Manager (SAM, University of Washington, Seattle, USA) [33] demonstrated improved compliance with beta blocker and glucose management protocols [33,34], reduced gaps in blood pressure monitoring [33,35] as well as the duration and frequency of hypotension [36], and even suggested some cost reductions may be feasible [33]. In other cases, intraoperative CDS has shown similar benefits for antibiotic administration and clinical documentation [37]. However, improvements in process measures such as protocol compliance do not necessarily translate into improved outcomes [34,38]. Furthermore, developing such systems presents many challenges, including the need to demonstrate outcome improvements, impact on patient safety, conformance with regulatory requirements [39], and that some improvements may be more easily achieved through the relatively simpler implementation of *post hoc* reporting [29,40].

### Limitations

While the evaluation of the *telePORT* app was terminated due to an insurmountable technical issue, several shortcomings of the study should be noted prior to any further work on this or a similar initiative. A summative evaluation of the app should have included certain dimensions that we are not able to report here. A structured pre and postimplementation survey of all relevant stakeholders, including the anesthesiologists, may have provided useful opinions on the benefits of the app and process improvements. It might also have been possible to establish a quantifiable measure of an AA’s situational awareness based on their timing on response to critical events. This should include time to provide meaningful assistance as well as arrival, so it would require a careful and agreed upon definition.

We did not obtain a record of the reasons for app messaging (calls for help), including any occurrence of false alarms, which would provide another useful measure of the app’s utility. Finally, while no adverse events related to the use of the app were logged in our institutions’ patient safety and learning system, we also did not implement any measures to track vigilance of the app or resulting clinical errors. Taking this project forward would require a clear definition of the potential points of impact on the existing model of care that can be evaluated postimplementation.

### Future Work

Apps like *telePORT* and VigiVU have the potential to tighten integration with electronic anesthesia records and to support decision support tools, scheduling systems and dashboards to improve perioperative data flow and team communication, with bidirectional data feeds. It should be possible to modify *telePORT* to use Apple Push Notifications using cellular data to allow better connectivity, and to make it available in the Apple Store, so all anesthesia team members can use it with a (secure) bring-your-own-device model.

The *telePORT* app was designed for a small group of AAs supporting a larger number of anesthesiologists, but it also has potential as a portable tool for a single anesthesiologist monitoring multiple operating rooms or procedural suites, possibly supporting trainees or other care providers. In either case, an important addition to the tool’s functionality would be the enabling of closed-loop communication via confirmation that a message had been received.

As a next step, our research group has worked closely with pediatric critical care clinicians to develop and evaluate a preliminary, low-fidelity prototype of the VitalPAD, an app designed to improve the efficiency of clinical decision making, communication, and patient safety in the ICU [41]. The app will ultimately combine information from multiple monitoring and therapeutic devices in a single mobile app, which will include a map overview of the ICU showing clinician assignment, patient status and respiratory support, along with functions for display of patient vital signs, photo-documentation of ABG results, team communication and reminders.

The *telePORT* app and related projects by other research teams show the potential for innovative, clinically relevant, real-time applications. The research community would benefit greatly from an open protocol for vital signs streaming that would enable new intelligent apps to be rapidly developed and deployed in the hospital setting, with immediate benefits to healthcare professionals, researchers and patients. Protocols developed for the Internet of Things may offer a solution that already has strong community support [11].

### Conclusion

We demonstrated that day-to-day activities of the anesthesia team can be supported by a mobile app that integrates data collected in real-time from the OR monitors with a facility for anesthesiologists to send requests for help, as well as team communication features. We overcame significant technical challenges, benefitted from the use of user-centered design, and were able to demonstrate feasibility with the AAs at our institution. Issues with the local wireless network at our site prevented full-scale implementation, but the VitalNode server infrastructure, which collects and stores OR data, continues to benefit ongoing research and QI initiatives.

## Abbreviations

AA: anesthesia assistant
ABG: arterial blood gas
AIMS: anesthesia information management system
BCCH: British Columbia Children’s Hospital
CDS: clinical decision support
CPU: central processing unit
CSV: comma-separated values
CT: computed tomography
DHCP: dynamic host configuration protocol
ECG: electrocardiogram
etCO_2_: end-tidal carbon dioxide
HR: heart rate
ICU: intensive care unit
IP: internet protocol
IQR: interquartile range
MRI: magnetic resonance imaging
NIBP: noninvasive blood pressure
OR: operating room
PCAP: packet capture
QI: quality improvement
RR: respiratory rate
SAM: smart anesthesia manager
SpO_2_: blood oxygen saturation
telePORT app: Portable Operating Room Tracker app
VoIP: Voice over Internet Protocol
